# Interferon signaling drives epithelial metabolic reprogramming to promote secondary bacterial infection

**DOI:** 10.1371/journal.ppat.1011719

**Published:** 2023-11-08

**Authors:** Grace P. Carreno-Florez, Brian R. Kocak, Matthew R. Hendricks, Jeffrey A. Melvin, Katrina B. Mar, Jessica Kosanovich, Rachel L. Cumberland, Greg M. Delgoffe, Sruti Shiva, Kerry M. Empey, John W. Schoggins, Jennifer M. Bomberger

**Affiliations:** 1 Department of Microbiology and Molecular Genetics, University of Pittsburgh School of Medicine, Pittsburgh, Pennsylvania, United States of America; 2 Department of Microbiology and Immunology, Geisel School of Medicine at Dartmouth, Hanover, New Hampshire, United States of America; 3 Department of Microbiology, University of Texas Southwestern Medical Center, Dallas, Texas, United States of America; 4 Department of Pharmacy and Therapeutics and Center for Clinical Pharmaceutical Sciences, University of Pittsburgh School of Pharmacy, Pittsburgh, Pennsylvania, United States of America; 5 Department of Immunology, University of Pittsburgh School of Medicine, Pittsburgh, Pennsylvania, United States of America; 6 Department of Pharmacology and Chemical Biology and Vascular Medicine Institute, University of Pittsburgh School of Medicine, Pittsburgh, Pennsylvania, United States of America; University of Maryland, UNITED STATES

## Abstract

Clinical studies report that viral infections promote acute or chronic bacterial infections at multiple host sites. These viral-bacterial co-infections are widely linked to more severe clinical outcomes. In experimental models *in vitro* and *in vivo*, virus-induced interferon responses can augment host susceptibility to secondary bacterial infection. Here, we used a cell-based screen to assess 389 interferon-stimulated genes (ISGs) for their ability to induce chronic *Pseudomonas aeruginosa* infection. We identified and validated five ISGs that were sufficient to promote bacterial infection. Furthermore, we dissected the mechanism of action of *hexokinase 2* (*HK2*), a gene involved in the induction of aerobic glycolysis, commonly known as the Warburg effect. We report that *HK2* upregulation mediates the induction of Warburg effect and secretion of L-lactate, which enhances chronic *P*. *aeruginosa* infection. These findings elucidate how the antiviral immune response renders the host susceptible to secondary bacterial infection, revealing potential strategies for viral-bacterial co-infection treatment.

## Author summary

Viral infections can make the host susceptible to secondary bacterial infections. Increasing evidence indicates that viral-bacterial co-infections cause worse clinical manifestations and lead to life-threatening acute or chronic bacterial infections. The host immune response against a viral infection promotes secondary bacterial infection; however, our understanding of how the antiviral response favors a subsequent bacterial infection is limited. Here, we aimed to assess the role of a suite of genes induced by the antiviral response, known as interferon-stimulated genes (ISGs), in the enhancement of *Pseudomonas aeruginosa* infection, which is frequently found in viral-bacterial co-infections in the airways of immunocompromised individuals. We found that the ISG *hexokinase 2* (*HK2)*, which drives metabolic reprogramming and mediates the secretion of the metabolite lactate by the respiratory epithelium, promotes chronic *P*. *aeruginosa* infection. These findings improve our understanding of how the antiviral response changes the host mucosal environment, enabling a subsequent bacterial infection.

## Introduction

Increasing evidence shows that viral infections render the host susceptible to secondary bacterial infection in multiple host sites. The clinical manifestations of viral infections vary from mild, with symptoms that resolve within days, to more severe in cases that require hospitalization. Viral-bacterial co-infections are often linked to worsened outcomes, leading to extended stays in intensive care units, with prolonged and more severe clinical symptoms [1,2]

Viral infections predispose to the acquisition of acute or chronic bacterial infections. They are associated with multiple diseases in the airways [3–9], the auditory cavity [10], and the urinary [11] and gastrointestinal tracts [12–15]. Moreover, they have been linked to severe conditions such as meningitis and sepsis [11]. Clinical studies have shown that influenza virus and respiratory syncytial virus (RSV) predispose the airways to infection by *Streptococcus pneumoniae*, *Haemophilus influenzae*, *Moraxella catarrhalis* and *Staphylococcus aureus* in children and adults [4–6,8,16,17] and these co-infections are associated with more severe symptoms [9,18,19]. Moreover, in adults and those suffering from chronic lung disease, RSV has been shown to predispose to chronic *Pseudomonas aeruginosa* infection [3,7,9,18,19]. While clinical studies examining viral-bacterial co-infections have become more common, there remains limited research about the mechanisms by which viral infections predispose to secondary bacterial infection and, in particular, how the antiviral immune response promotes co-infection.

Previous work by our laboratory and others has linked a preceding viral infection and subsequent antiviral immune responses to an increased likelihood of secondary bacterial infection [20–23]. Studies in an *in vivo* model of airway infection have shown that the antiviral response to different respiratory viruses impairs the innate immune control of *Streptococcus pneumoniae a*nd leads to an increased bacterial burden in the nasal cavity [20]. Similarly, we have previously shown that the antiviral immune response enhances chronic *Pseudomonas aeruginosa* infection in an *in vitro* model that closely mimics the respiratory tract [22,23]. Although viral-bacterial co-infection models have been used to show that antiviral interferon signaling is detrimental to the clearance of bacterial infections, the underlying mechanisms remain unknown.

Viral infections trigger an antiviral response that reshapes the host intracellular and extracellular environment. This antiviral response involves a signaling cascade that drives increased expression of hundreds of interferon-stimulated genes (ISGs), some of which have antiviral effector functions [24,25]. The development and implementation of high-throughput ISG screens have allowed the identification of the antiviral functions of hundreds of ISGs by multiple viruses [26,27]; however, their role in viral-bacterial co-infections has yet to be tested.

To define mechanisms by which antiviral interferon signaling promotes bacterial infections, specifically through the induction of ISGs, we implemented a lentivirus-based ISG screen to identify ISGs that promote chronic *P*. *aeruginosa* infection in the respiratory tract. We identified 5 hit ISGs and further dissected the mechanism by which one hit, *hexokinase 2* (*HK2*), stimulates *P*. *aeruginosa* biofilm formation. *HK2* encodes one of the first rate-limiting enzymes in glycolysis. The enzyme HK2 is predominantly upregulated in cells undergoing aerobic glycolysis, also known as the Warburg effect (WE). This process is linked to a higher demand for glucose to supply mitochondrial respiration and shunts the excess glucose toward the synthesis of L-lactate, which is ultimately secreted and thus accessible in the extracellular milieu [28–31]. Our results suggest that the antiviral interferon response is the key driver of this metabolic reprogramming that supports secondary bacterial infections and thus contributes to transkingdom interactions.

## Results

### Identification of ISGs that promote *P*. *aeruginosa* biofilm growth

**Fig 1:**
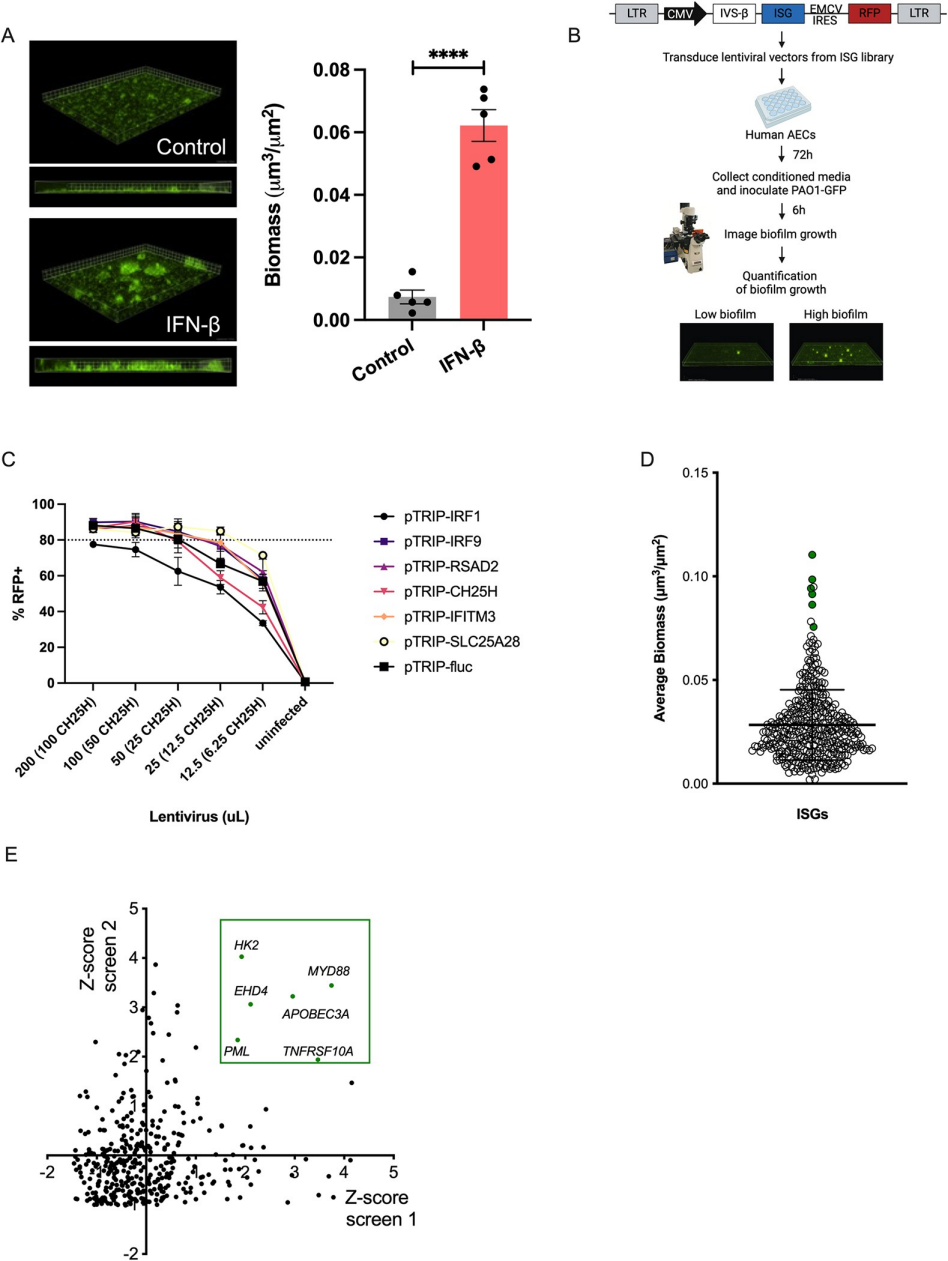
Identification of ISGs that promote *P*. *aeruginosa* biofilm growth. (A) Biofilm biomass of GFP-expressing *P*. *aeruginosa* grown in apical secretions from IFN-β-stimulated CF AECs determined using a static abiotic biofilm assay. Representative fields of 3 independent replicates (left) and quantification of biofilm biomass using Nikon NIS-Elements from images acquired at 20x (right). (B) Diagram of ISG overexpression screen and biofilm biomass quantification created with Biorender.com. (C) Transduction efficiency using different lentivirus doses, with 50 uL of lentiviral particles as the lowest dose reaching 80% transduction. (D) Average of *P*. *aeruginosa* biofilm biomass per ISG in 2 independent screens represented as a dot plot, highlighting ISGs with the highest biomass increase (green dots) measured under abiotic conditions. (E) PCA of screen 1 and 2 highlighting 6 ISGs with a Z-score ≥2 (in green). The 6 ISGs hits are *TFRSF10A*, *PML*, *APOBEC3A*, *MYD88*, *EHD4* and *HK2*. For all experiments n *≥* 3. Data are presented as mean *±* SEM. ****p < 0.0001.

We have previously demonstrated that respiratory syncytial virus (RSV) infection increases *P*. *aeruginosa* biofilm growth through IFN signaling [22]. To define mechanisms by which antiviral interferon signaling enhances *P*. *aeruginosa* biofilm growth, we hypothesized that respiratory epithelial cells secrete biofilm-stimulatory factors. To test this hypothesis, human ΔF508/ΔF508 cystic fibrosis airway epithelial cells (CFBE41o-, hereafter called CF AECs) were grown as a polarized monolayer and stimulated with IFN-β (1000 IU/mL). The secretions from the apical compartment were collected and inoculated with GFP-tagged *P*. *aeruginosa*. After allowing biofilm biogenesis, we imaged and quantified biofilm biomass via fluorescence microscopy. We observed an increase in *P*. *aeruginosa* biofilm biomass grown in the apical secretions of IFN-β-stimulated CF AECs compared to the unstimulated control (Fig 1A). To determine whether specific ISGs induce *P*. *aeruginosa* biofilm formation, we screened in duplicate a library of 389 ISGs using a previously described cell-based lentiviral system [26]. The lentiviral vector co-expresses an individual ISG and the red fluorescent protein TagRFP (Fig 1B). To test the transduction efficiency and validate our screening method, we transduced CF AECs with 2 genes that positively regulate the expression of ISGs (*IRF1* and *IRF9*) and 4 ISGs with known functions (*RSAD2*, *CH25H*, *IFITM3*, and *SLC25A28*). We selected the lowest lentivirus dose yielding 80% RFP/ISG-expressing+ cells (Fig 1C). The ISG screen included *P*. *aeruginosa* grown in minimal essential medium (MEM) as a negative control and inoculation of *P*. *aeruginosa* in the apical secretions of cells with lentiviral overexpression of the firefly luciferase (*Fluc)* gene as a control of transduction (S1 Fig). CF AECs were transduced with the lentiviruses expressing each ISG for 72 h. We incubated GFP-producing *P*. *aeruginosa* in apical secretions from each ISG and assessed biofilm growth. Using fluorescence microscopy, we imaged, quantified, and averaged 6 different fields of GFP-expressing *P*. *aeruginosa* biofilms grown in apical secretions for each ISG tested. From 2 biological screening replicates, we identified a set of ISGs inducing the highest biofilm biomass production compared to the global mean (Fig 1D). We then calculated a z-score based on the biomass obtained from each condition, and z-scores above 2 were considered to indicate significance. Conditions with a z-score of 2 in both screens were considered hits. We identified 6 hit ISGs that significantly enhanced *P*. *aeruginosa* biofilm growth: *TNF Receptor Superfamily Member 10a (TNRFSF10A*, *z score = 2*.*7)*, *PML Nuclear Body Scaffold (PML*, *z score = 2*.*1)*, *Apolipoprotein B MRNA Editing Enzyme Catalytic Subunit 3A (APOBEC3A*, *z score = 3*.*1)*, *MYD88 Innate Immune Signal Transduction Adaptor (MYD88*, *z score = 3*.*6)*, *EH Domain Containing 4 (EHD4*, *z score = 2*.*6)* and *Hexokinase 2 (HK2*, *z score = 3)* (Fig 1E).

### Verification of *TNFRSF10A*, *PML*, *APOBEC3A*, *MYD88*, *EHD4* and *HK2 as* ISG hits enhancing *P*. *aeruginosa* biofilm growth

**Fig 2:**
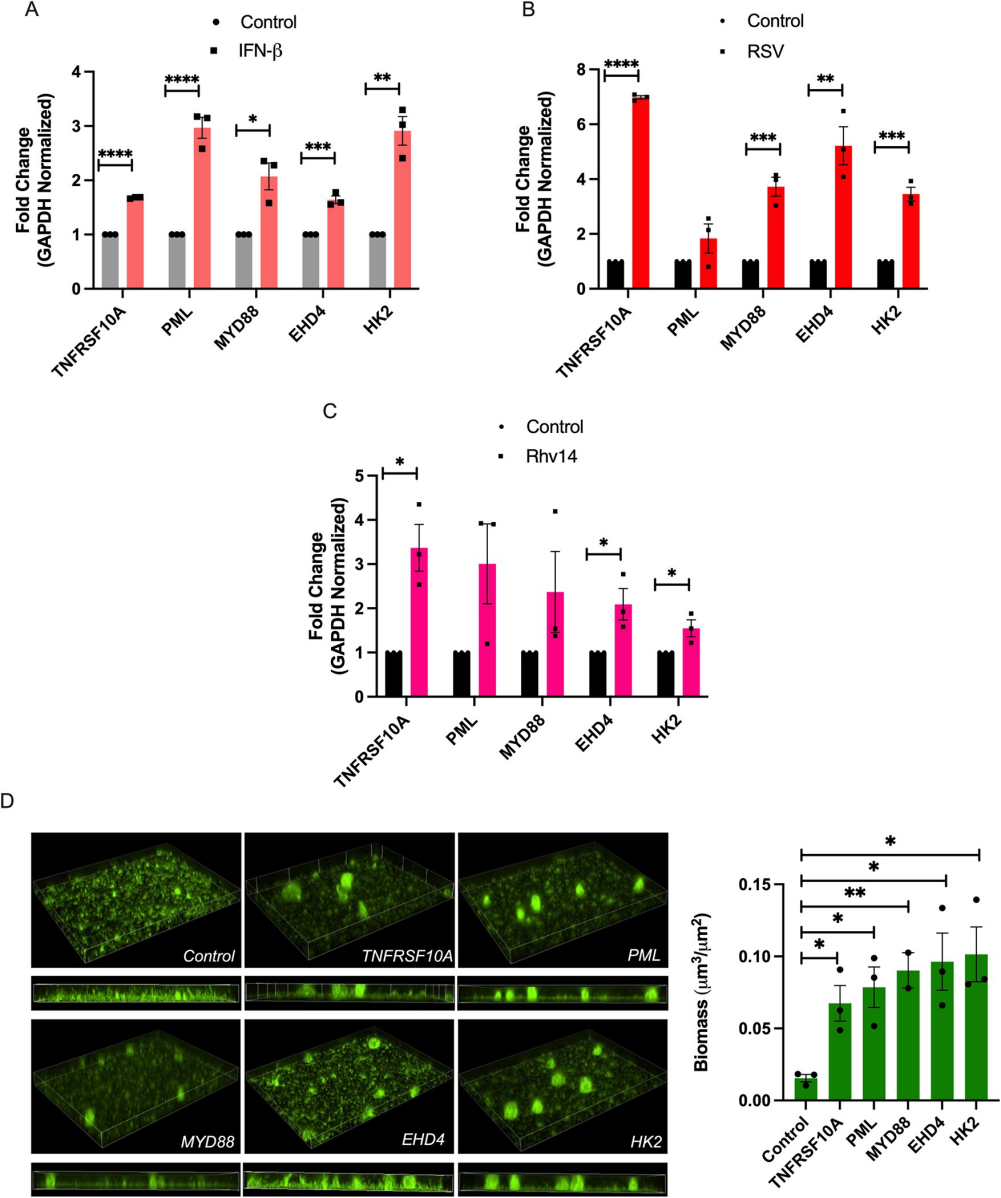
Confirmation of selected hit ISGs that stimulate *P*. *aeruginosa* biofilm formation. (A-C) mRNA fold change of *TNFRSF10A*, *PML*, *MYD88*, *EHD4* and *HK2* in IFN-β-stimulated (A), RSV-infected (B) or hRV-infected (C) CF AECs measured by quantitative RT-PCR (qRT-PCR). (D) Biofilm biomass of GFP-expressing *P*. *aeruginosa* grown in apical secretions from CF AECs overexpressing GFP (transfection control), *TNFRSF10A*, *PML*, *MYD88*, *EHD4* or *HK2* using liposome-mediated transfection and measured under static abiotic conditions. Biofilm biomass quantified with Nikon NIS-Elements from images acquired at 20x. For all experiments n *≥* 3. Data are presented as mean *±* SEM. *p < 0.05, **p < 0.01, ***p < 0.001, ****p < 0.0001.

With the identification of *TNFRSF10A*, *PML*, *APOBEC3A*, *MYD88*, *EHD4*, and *HK2* as ISGs that promote a significant increase of *P*. *aeruginosa* biofilm formation, we tested whether these 6 ISG hits were induced by IFN or respiratory viral infection. We used qRT-PCR to assess their expression in CF AECs stimulated with IFN-β or infected with two different respiratory viruses, RSV or human rhinovirus (hRV14). We observed upregulation of the 6 ISGs during IFN-β stimulation (Fig 2A) and during infection with either of the two respiratory viruses relevant to chronic lung disease patients (Fig 2B and 2C), indicating that their upregulation is mediated by IFN signaling.

We then implemented a secondary validation for the 6 ISG hits. We adapted an overexpression platform using liposome-mediated transfection of plasmid encoding each ISG hit in CF AECs. After transfection, we collected apical secretions to grow GFP-expressing *P*. *aeruginosa* biofilms for 6 h. We then imaged and quantified biofilm biomass by fluorescence microscopy. We observed induction of *P*. *aeruginosa* biofilm formation on CF AECs overexpressing *TNFRSF10A*, *PML*, *MYD88*, *EHD4*, and *HK2* (Fig 2D). We detected cytotoxicity triggered by *APOBEC3A* overexpression and did not further pursue this ISG hit in subsequent experiments. These results suggest that *TNFRSF10A*, *PML*, *MYD88*, *EHD4*, and *HK2* are induced during IFN signaling and that their upregulation provokes the secretion of molecule(s) that stimulate *P*. *aeruginosa* biofilm formation.

### RSV infection induces *hexokinase 2 (HK2)* through the antiviral response and triggers the Warburg effect

**Fig 3:**
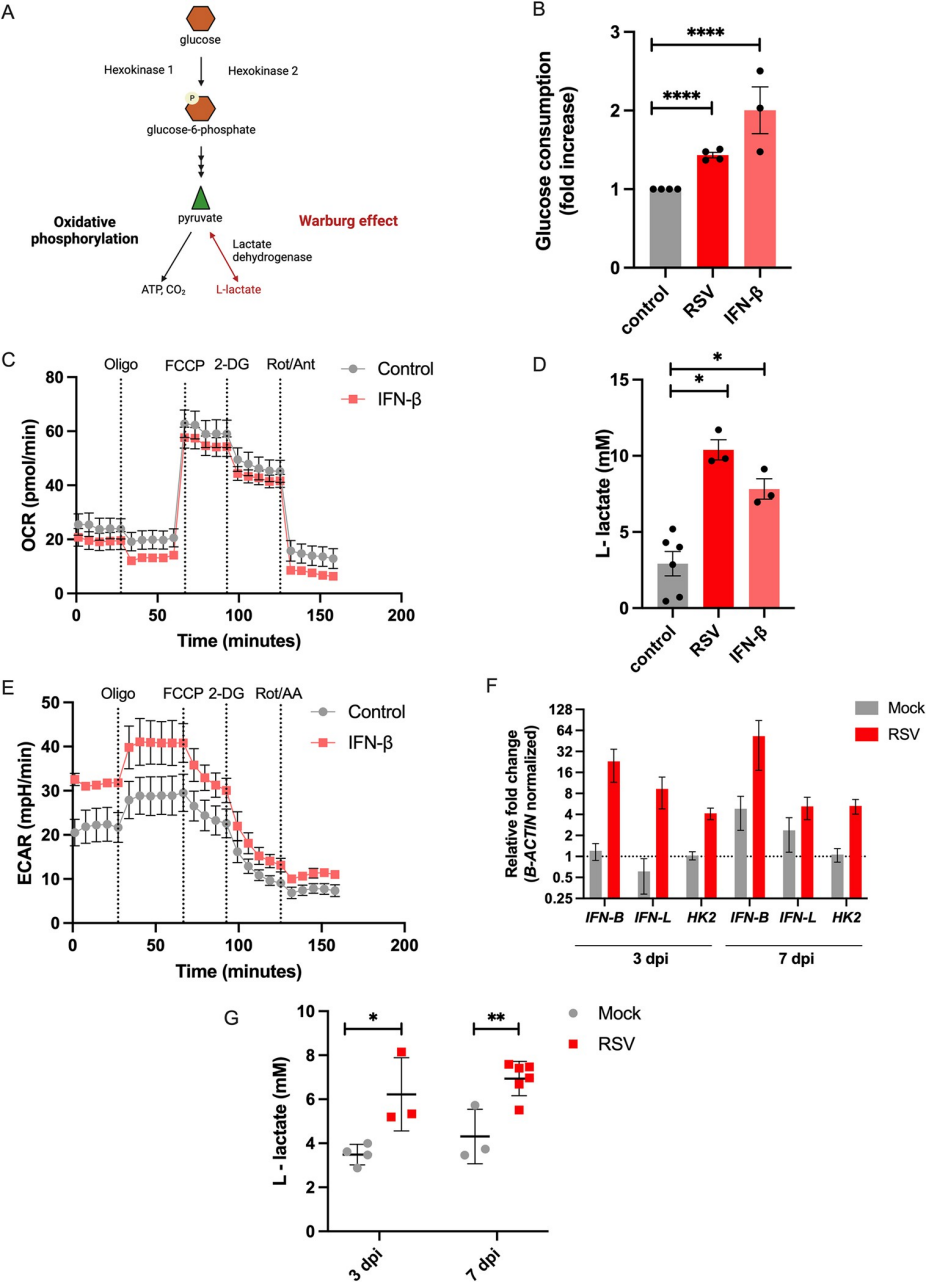
RSV infection induces *HK2* expression and triggers the Warburg effect in bronchial epithelial cells through the antiviral response. (A) Diagram of glycolysis depicting the fate of pyruvate toward oxidative phosphorylation or toward aerobic glycolysis (WE) created with Biorender.com. (B) Glucose consumption in the basolateral medium of IFN-β-stimulated or RSV-infected CF AECs measured by colorimetric assay. (C) OCR in CFBE41o- cells during IFN-β stimulation determined using Seahorse assay. (D) L-lactate concentration measured in apical secretions from CF AECs during IFN-β stimulation or RSV infection measured by colorimetric assay. (E) ECAR in IFN-β-stimulated CFBE41o- cells determined using Seahorse assay. Lung tissue and bronchoalveolar lavage fluid (BALF) were collected from BALB/cJ neonatal mice infected with line 19F RSV (5x10^5^ pfu/g) 3 and 7 days post-infection. (F) mRNA expression of *Ifn-B*, *Inf-L*, and *HK2*, expressed as fold change, was measured in lung tissue. (G) Quantification of the L-lactate concentration in BALF determined using colorimetric assay. For all experiments n *≥* 3. Data are presented as mean *±* SEM. *p < 0.05, **p < 0.01, ***p < 0.001, ****p < 0.0001.

We next investigated the mechanism by which the hit gene *HK2* promoted *P*. *aeruginosa* biofilm formation. *HK2* was prioritized because of the robust biofilm induction in the primary screen (z score = 3) and secondary validation (Figs 1D and 2D). *HK2* encodes the enzyme hexokinase 2 (HK2), one of the four hexokinase isoforms found in mammalian tissues. Hexokinases are responsible for the first rate-limiting step in glycolysis. HK1 is constitutively produced in most cell type and leads glycolysis toward the synthesis of pyruvate and its subsequent transport to the mitochondria, where it supports respiration and oxidative phosphorylation. In addition, cells with high glucose flux [30,31] also express HK2, which leads the excess of pyruvate toward the synthesis of L-lactate through a process known as aerobic glycolysis or Warburg effect (WE) [28,32] (Fig 3A). To determine if antiviral immune signaling triggers the WE, we stimulated CF AECs with IFN-β and measured the glycolytic status. We measured glucose consumption and oxygen consumption rate (OCR) to investigate how glycolysis supplies mitochondrial respiration. We observed increased glucose consumption under IFN-β stimulation (Fig 3B), in accordance with higher glycolytic flux commonly found in cells undergoing the WE. We also observed a similar OCR (Fig 3C) and basal respiration (S2A Fig) during IFN-β stimulation, indicating that glycolysis is feeding mitochondrial respiration. We additionally assessed glycolytic function during RSV infection. Likewise, we found increased glucose consumption (Fig 3B) and a similar trend in OCR and basal respiration (S3A and S3B Fig). Glucose consumption was also increased during hRV14 infection (S4A Fig). These results suggest that antiviral IFN signaling leads to higher glycolytic flux, which supports the WE and mitochondrial respiration.

We next measured the secretion of L-lactate and extracellular acidification rate (ECAR) to determine if IFN signaling is also driving glycolysis toward Warburg metabolism. We found higher lactate levels in secretions from IFN-β-stimulated CF AECs than in the unstimulated control (Fig 3D). We observed similar results in secretions from cells infected with RSV (Figs 3D and S3E) and hRV14 (S4B Fig), We also found an increased ECAR and enhanced basal glycolysis in cells under IFN stimulation (Figs 3E and S2B) and during RSV infection (S3C and S3D Fig), as measured by Seahorse mitochondrial stress analysis. To examine if *HK2* upregulation was sufficient to cause this metabolic shift, we overexpressed *HK2* in AECs and examined lactate secretion. We observed that *HK2* overexpression induces L-lactate secretion, suggesting that *HK2* upregulation is sufficient to promote lactate secretion (S5 Fig). Overall, these data suggest that viral-induced IFN signaling leads to an increase in glucose consumption and apical L-lactate secretion in bronchial epithelial cells, consistent with IFN-induced *HK2* reprogramming the metabolic state of the respiratory epithelium to the WE.

Next, we wanted to determine if our *in vitro* observations of antiviral IFN signaling inducing the WE could be recapitulated in an *in vivo* model. We infected neonatal BALB/cJ mice with 5x10^5^ pfu/g line 19F RSV [33] and collected lung tissue and bronchoalveolar lavage fluid (BALF) at 3 and 7 days post-infection (dpi). We found increased expression of *Ifn-B*, *Ifn-L*, and *HK2* at both time points compared to that in the uninfected control, which is consistent with the induction of the antiviral response along with the upregulation of the glycolytic gene *HK2* (Fig 3F). We next measured L-lactate secretion in BALF collected at 3 and 7 dpi. We found increased levels of L-lactate in RSV-infected mice compared to that in the controls (Fig 3G). These results suggest that RSV infection induces Warburg metabolism *in vivo*. Taken together, these findings demonstrate that the IFN signaling induced during a viral infection triggers the WE.

### Lactate secreted by the CF bronchial epithelium stimulates *P*. *aeruginosa* biofilm growth

**Fig 4:**
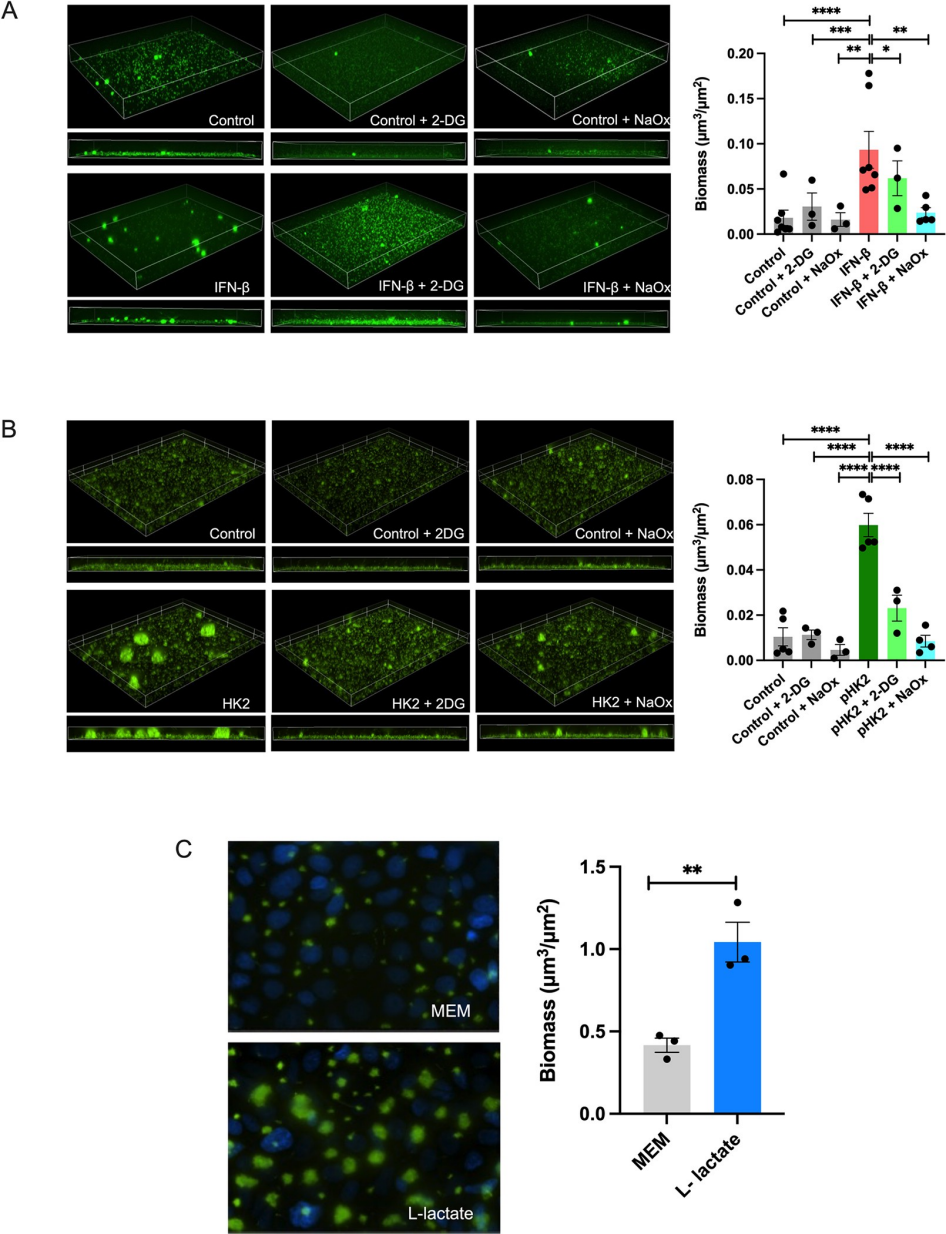
L-lactate stimulates *P*. *aeruginosa* biofilm growth. (A, B) Biofilm biomass of GFP-expressing *P*. *aeruginosa* grown in apical secretions from IFN-β-stimulated CF AECs (A) or grown in apical secretions from HK2-overexpressing CF AECs (B) treated with 2-DG or NaOx and assessed by a static abiotic biofilm assay. *HK2* overexpression was induced through liposome-mediated transfection of a plasmid encoding *HK2*. Representative fields of 3 independent replicates (left) and quantification of biofilm biomass using Nikon NIS-Elements with images acquired at 20x (right). (C) Biofilm biomass of GFP-producing *P*. *aeruginosa* co-cultured with CFBE41o- cells under constant flow with L-lactate supplementation assessed under biotic conditions. Biofilms (green) and nuclei stained with Hoechst (blue) were imaged by live-cell microscopy (left), and biofilm biomass was quantified by Nikon NIS-Elements (right). For all experiments n *≥* 3. Data are presented as mean *±* SEM. *p < 0.05, **p < 0.01, ***p < 0.001, ****p < 0.0001.

After observing that *HK2* upregulation by IFN signaling triggers the WE both *in vitro* and *in vivo*, we examined the impact of epithelial metabolic reprogramming on *P*. *aeruginosa* biofilm biogenesis. To this end, we used pharmacological inhibitors of the WE during IFN-β stimulation or *HK2* overexpression. We used 2-deoxy-glucose (2-DG), an analogue of glucose, to inhibit HK2 function and sodium oxamate (NaOx), an analogue of pyruvate, to inhibit the conversion of pyruvate to L-lactate. We treated CF AECs with either 2-DG (10 mM) or NaOx (50 mM), collected apical secretions, and inoculated *P*. *aeruginosa* into those secretions for biofilm biomass quantification. We observed that secretions from IFN-β-stimulated CF AECs enhanced *P*. *aeruginosa* biofilm formation compared to that of the unstimulated control, with a reduction in *P*. *aeruginosa* biofilm biomass when IFN-β-stimulated CF AECs were treated with 2-DG or NaOx (Fig 4A). We further used *HK2*-overexpression to determine whether HK2 induction alone is sufficient for inducing *P*. *aeruginosa* biofilm formation. We observed that while apical secretions from *HK2*-overexpressing CF AECs enhanced *P*. *aeruginosa* biofilm biomass, the presence of each of these glycolytic inhibitors led to a decrease in *P*. *aeruginosa* biofilm biomass to untreated levels (Fig 4B). These findings suggest that inhibiting the synthesis of L-lactate and its apical secretion impairs *P*. *aeruginosa* biofilm growth.

Finally, we wanted to determine whether growing *P*. *aeruginosa* in the presence of L-lactate is sufficient to induce biofilm formation. To this end, we used a live-cell imaging system in which CF AECs are co-cultured with GFP-producing *P*. *aeruginosa* for 6 h under a constant flow of medium supplemented with L-lactate (10 mM) [22,34,35]. We used L-lactate, as it is the isomeric form synthesized in mammalian cells and applied the concentration released during RSV infection. We next imaged biofilms grown on CF AECs by fluorescence microscopy and quantified the biofilm biomass. We observed enhanced *P*. *aeruginosa* biofilm formation in medium supplemented with L-lactate compared to that in the untreated control (Fig 4C), suggesting that lactate secreted by the airway epithelium enhances *P*. *aeruginosa* biofilm formation. Overall, these findings are consistent with the conclusion that IFN signaling stimulates the expression of the ISG *HK2*, which drives metabolic reprogramming and the WE, stimulates apical L-lactate secretion, and thereby enhances *P*. *aeruginosa* biofilm growth.

## Discussion

The expression of ISGs during viral infection leads to changes in the intracellular environment, thus regulating the host-virus crosstalk. Although the functions of many ISGs have been studied in the context of viral infections, little is known about the functions of virus-induced ISGs in the setting of viral-bacterial co-infections, in which IFN signaling is known to potentiate secondary bacterial infections. Here, we screened 389 ISGs previously described to regulate multiple viral infections [26], and we identified 5 hit ISGs, namely, *TNRFSF10A*, *PML*, *MYD88*, *EHD4* and *HK2*, that robustly promoted *P*. *aeruginosa* biofilm growth. Moreover, we dissected the mechanism of *HK2* in enhancing *P*. *aeruginosa* biofilm growth, showing that it instigates aerobic glycolysis and the apical secretion of L-lactate, which thereby potentiates *P*. *aeruginosa* biofilm growth. Taken together, our findings support a model in which antiviral signaling mediates metabolic reprogramming in the airway epithelium, which stimulates *P*. *aeruginosa* biofilm biogenesis. Finally, this study demonstrates that applying high-throughput based ISG screens, previously used to screen single viral [26] or bacterial [36] infections offers a valuable tool to study viral-bacterial co-infections, furthering our understanding of transkingdom interactions during co-infection.

Although ISGs are canonically referred to as antiviral effectors, the upregulation of *HK2* mainly has a proviral role among respiratory viral infections [37–40]. Multiple viruses enhance glucose metabolism in the host, even in the presence of oxygen to supply mitochondrial respiration, thus resembling the WE widely studied in cancer, also known as aerobic glycolysis. Although the main role of aerobic glycolysis appears to be the rapid generation of ATP, it also funnels metabolites to other pathways including the pentose phosphate pathway (PPP) for the synthesis of nucleosides, fatty acids and antioxidant molecules and the hexosamine biosynthetic pathway (HBP) that supports the generation of metabolites for protein and lipid glycosylation [28,29,32]. As intracellular parasites, viruses rely on the host cell machinery and resources for viral replication; thus, it is not surprising that aerobic glycolysis plays a key role in the success of viral infection. *HK2* encodes the first rate-limiting enzyme of aerobic glycolysis, and it is upregulated during infections caused by multiple respiratory viruses including RSV, influenza virus, human metapneumovirus (hMPV) and human rhinovirus (hRV) [37–41]. Interestingly, *HK2* has a proviral function in RSV, influenza virus, hMPV and adenovirus 5 infection [37–41] but exerts an antiviral function in hRV-C infection [41], revealing some virus-specific roles. Our results show for the first time that the induction of HK2 is the result of the host antiviral immune program, i.e., the IFN response.

A hallmark feature of the WE is the secretion of lactate in the extracellular environment, and recently, the role of lactate in host-pathogen interactions is increasingly appreciated after long being considered a waste product of glucose metabolism. Interestingly, it was recently proposed that lactate produced during virus-induced aerobic glycolysis inhibits RIG-I signaling, thus suppressing RIG-I-mediated IFN synthesis and allowing viral replication [42]. This hypothesis suggests that HK2-driven aerobic glycolysis supports viral infections by providing energy and biomolecules for replication, as well as by providing lactate as a metabolite to evade host defense.

Lactate exists as two enantiomers, L- and D-lactate. While L-lactate is the main form found in vertebrates as a byproduct of either anaerobic or aerobic glycolysis, D-lactate is mainly synthesized by bacteria under anaerobic conditions [43]. It is well documented that L-lactate is present at high levels at sites of inflammation [44,45]. And notably, clinical studies of acute or chronic infection and inflammation in the lungs have shown a predominance of the L-lactate form, suggesting that the host, specifically innate immune cells and the epithelium, is the main source of this glycolytic metabolite [41–45].

Although the role of L-lactate in viral-bacterial co-infections is unknown, increasing evidence from research on acute and chronic infections in the airways suggests an association between viral infection, increased L-lactate levels, and secondary bacterial infection. Clinical evidence from airway samples and studies on *in vitro* models of the respiratory epithelium have shown that viral infections increase the concentration of L-lactate [44]. In addition, some clinical studies have linked respiratory viral infections and higher levels of L-lactate with episodes of pulmonary exacerbation, characterized by worsened lung symptoms, including worsened bacterial infection [44,46]. Here, using a model of viral-bacterial co-infection in the airways, we demonstrate that the antiviral IFN response promotes secondary *P*. *aeruginosa* infection through the upregulation of the ISG *HK2*, which is required for the WE and apical L-lactate secretion. Overall, these findings suggest that *P*. *aeruginosa* uses lactate secreted from the airway epithelium during the antiviral response to promote biofilm formation.

Multiple bacteria that infect the airways, including *P*. *aeruginosa*, *Neisseria meningitidis* and *Haemophilus influenzae*, use host-derived lactate for colonization and persistence [47–55]. In studies of the CF airways, it has been found that lactate in CF sputum and synthetic CF sputum medium (SCFM) is among a diverse group of carbon sources used by *P*. *aeruginosa*, highlighting that *P*. *aeruginosa* can respond to the nutritional cues of the CF lung environment [50,51]. Growing a clinical isolate of *P*. *aeruginosa* in medium supplemented with lactate led to a genetic profile associated with the biofilm lifestyle, inducing the upregulation of genes involved in phenazine production and downregulation of genes involved in swarming motility [52]. Interestingly, in our study we observed that L-lactate stimulates *P*. *aeruginosa* biofilm biogenesis. Future work will examine the underlying mechanism by which *P*. *aeruginosa* responds to L-lactate to induce biofilm. In summary, the host-derived L-lactate produced during the antiviral immune response plays a key role in promoting secondary bacterial infection.

A majority of the research on viral-bacterial co-infections has focused on how the antiviral response renders the host susceptible to secondary bacterial infections. However, fewer studies point out that having a bacterial infection first can also predispose the host to a secondary viral infection [56–59]. Recent studies suggest that *S*. *pneumoniae* colonization promotes the infectivity and transmission of influenza [59]. In addition, previous work from our laboratory demonstrated that *P*. *aeruginosa* secretes at least one virulence factor that reduces viral antigen presentation and recognition of influenza-infected respiratory epithelial cells [60]. However, more studies are necessary to determine if immunometabolic regulation is part of a broadly applicable mechanism by which bacterial infections can predispose or impact the severity of viral infections.

While IFN signaling is known to be detrimental to the host defense against a variety of extracellular pathogens, the mechanisms underlying this dysfunction remain poorly understood. The studies herein have identified the downstream ISGs *TNRFSF10A*, *PML*, *MYD88*, *EHD4*, and *HK2* that potentiate secondary bacterial infection. The function of these ISGs are in different cellular functions, including cell death and survival [61], nuclear bodies structure [62], inflammatory signaling [63], endocytic trafficking [64] and glucose metabolism [65]. While no apparent overlap in cellular functions suggest synergy of these ISGs in promoting biofilm, further studies are required to identify whether they act synergistically or through independent mechanisms to promote biofilm. Here, we uncovered the molecular mechanism by which *hexokinase 2* mediates biofilm formation due to metabolic reprogramming in the host. By elucidating the role of specific pathways downstream of the antiviral IFN response that promote secondary bacterial infection, we might identify new host targets with therapeutic potential to combat the poor outcomes observed, particularly in chronic lung disease patients.

## Materials and methods

### Ethics statement

*In vivo* assays strictly followed the recommendations of the NIH *Guide for the Care and Use of Laboratory Animals* [56] and were approved by the University of Pittsburgh Institutional Animal Care and Use Committee (IACUC) (protocol number 14023340). Neonatal BALB/cJ mice were handled according to IACUC guidelines, and all efforts were made to minimize animal suffering. Mice were housed at the University of Pittsburgh Division of Laboratory Animal Resources.

### Cell lines, viruses, and bacterial strains

We used the immortalized human CF airway epithelial cell line CFBE41o- (referred to herein as CF AECs) collected from explanted lungs of CF patients, following the protocol approved by the Institutional Review Board at the University of Pittsburgh [35]. CFBE41o- cells were seeded on Transwell filters and grown at air-liquid interface for 7–10 days or until well polarized or differentiated. For viral infections, CF AECs were apically infected with purified RSV A2 strain (MOI = 1) resuspended in minimal essential medium (MEM) without phenol red for 72 h at 37°C and with 5% CO_2_. For bacterial infections, GFP-expressing *Pseudomonas aeruginosa* strain PAO1 was used as previously described [22].

### Glycolytic inhibition experiments

2-Deoxy-glucose (2-DG, Sigma D8375) and sodium oxamate (NaOx, Sigma 02751) were purchased from Sigma Aldrich. For glycolytic inhibition during IFN-β stimulation, either 10 mM 2-DG or 50 mM NaOx was added to CF AECs basolaterally for 2 h at 37°C and with 5% CO_2_. The basolateral medium was replaced with medium supplemented with IFN-β (1000 IU/mL, R&D 8499-IF-010/CF), MEM without phenol red was added apically, and apical secretions were collected after 18 h for static abiotic biofilm formation experiments. For glycolytic inhibition during *HK2* overexpression, 48 h post-transfection, cells were treated with either 10 mM of 2-DG or 50 mM NaOx as previously described, and MEM without phenol was added apically. Apical secretions were collected 72 h post-transfection.

### ISG screen

The ISG library and the generation of the lentiviral particles were previously described [26]. Briefly, a library of 389 ISG was compiled based on microarrays of IFN-treated cells, and each ISG was inserted in a bicistronic lentivirus co-expressing the ISG and the red fluorescent protein TagRFP. Transduction efficiency was visually assessed as the percentage of cells that were RFP+. CF cells were spinoculated with equal volumes of lentivirus-containing supernatants in a one gene per well format at 800 *xg* for 45 minutes at 37°C. Lentivirus was removed at 6 hours post-transduction. ISG-conditioned supernatants were subsequently collected 48 hours post-transduction and maintained at 4°C. Each ISG-conditioned supernatant was added to a glass-bottomed 48 well plate and inoculated with PAO1 (OD_600_ = 0.01) for 6 h. Biofilms formed were imaged at 20x, and the biofilm biomass was quantified with a Nikon Ti inverted fluorescence microscope using Nikon Imaging Software (NIS) Elements AR 4.6. The ISG screen included PAO1 grown in MEM as negative control, PAO1 grown in MEM supernatant from cells overexpressing the *Fluc* gene, and PAO1 grown in supernatant from cells without lentiviral transduction, as transduction controls.

### Abiotic biofilm assay

GFP-expressing PAO1 (OD of the culture normalized to 0.05) was inoculated in apical secretions of CF AECs supplemented with 0.4% L-arginine in glass-bottomed dishes (MatTek Corporation), and biofilm formation was measured after 6 h of growth at 37°C and with 5% CO_2_. Z-stack images were recorded from 6–10 random fields per dish using a Nikon Ti-inverted fluorescence microscope. The area (μm^2^) and biofilm volume (μm^3^) of each Z-stack was measured using NIS Elements AR 4.6, and the biofilm biomass was determined by dividing the biomass volume/area (μm^3^/μm^2^).

### Mitochondrial function and aerobic glycolysis assessment

The OCR and ECAR were assessed using the Seahorse platform (Agilent) following 18 h of IFN-β stimulation or 24 h of RSV infection (MOI = 1). The OCR (pmol/minute) and ECAR (mpH/minute) were measured in basal conditions (medium alone) and after the sequential addition of oligomycin, FCCP, 2-DG, and the combination of rotenone and antimycin between 90–120 minutes. Glucose consumption (Abcam, ab65333) and lactate secretion (Abcam, ab65330) were measured after 18 h of IFN-β stimulation or 72 h of RSV infection using colorimetric assays. Glucose consumption was determined by subtracting the glucose concentration in basolateral medium from the glucose concentration in the feeding medium. For lactate secretion, MEM without phenol red was added apically for 18 h during IFN-β stimulation or for the last 24 h of RSV infection and was collected to determine the extracellular lactate concentration.

### Biotic biofilm imaging

CFBE410- cells under the constant flow of medium with 10 mM L-lactate (Sigma Aldrich, L7022) were inoculated with PAO1, and biofilm growth was recorded through a previously described live-cell imaging system [22, 35]

### Quantitative real-time PCR

RNA was extracted from CF AECs or neonatal BALB/cJ mice using a Qiagen RNA extraction kit (RNeasy Mini Kit, 74106) following the manufacturer instructions. PCR assays were run in a BioRad CFX connect system, and RNA expression was determined using the 2^-ΔΔ*CT*^ method.

### *In vivo* RSV infection

*In vivo* RSV assays strictly followed the recommendations of the NIH *Guide for the Care and Use of Laboratory Animals* [56] and were approved by the University of Pittsburgh Institutional Animal Care and Use Committee (IACUC) (protocol number 14023340). Neonatal BALB/cJ mice were infected intranasally with 5 × 10^5^ pfu/g body weight RSV line 19 or an equal volume of PBS, as previously described [22, 33]. Bronchoalveolar lavage fluid was collected to measure L-lactate levels, and lungs were harvested to quantify RNA expression.

### Statistical analysis

GraphPad Prism version 6.0 (GraphPad) was used for statistical analysis. Means were compared using Student’s *t* test or, for multiple comparisons, ANOVA with Tukey’s post hoc test. *p* < 0.05 was considered significant.

## Supporting information

S1 Fig ISG screen controls for *P*. *aeruginosa* biofilm growth. Representative images of GFP-producing P. aeruginosa biofilms under abiotic conditions grown in MEM (negative control), secretions from cells overexpressing *FLUC* (transduction control), and secretions from cells transfected with an ISG that resulted in low biofilm formation induction (*SLC16A1*) and an ISG that resulted in high biofilm formation induction (*CPT1A*). 20x images captured with Nikon NIS-Elements.
(TIF)

S2 Fig IFN-β signaling triggers non-mitochondrial glucose consumption. Measurement of (A) basal respiration and (B) basal glycolysis in CFBE41o- stimulated with IFN-β (1000 IU/mL) using a Seahorse assay. For all experiments n *≥* 3. Data are presented as mean *±* SEM. *p < 0.05.
(TIF)

S3 Fig RSV induces the expression of the ISG *HK2* and triggers the Warburg effect in bronchial epithelial cells. (A) OCR, (B) basal respiration, (C) ECAR, and (D) basal glycolysis measured in RSV-infected CFBE41o- cells using a Seahorse assay. (E) L-lactate concentration in apical secretions from primary CF AECs during RSV infection measured by colorimetric assay. For all experiments n *≥* 3. Data are presented as mean *±* SEM. *p < 0.05, **p < 0.01.
(TIF)

S4 Fig hRV14 infection induces glucose consumption and apical L-lactate secretion in CF AECs. Measurement of (A) glucose consumption in growth medium and (B) L-lactate concentration in apical secretions of CF AECs infected with hRV14 (MOI 0.1) for 72 h using a colorimetric assay. For all experiments n *≥* 3. Data are presented as mean *±* SEM. *p < 0.05, **p < 0.01.
(TIF)

S5 Fig HK2-overexpression in CF AECs enhances L-lactate synthesis and apical secretion. Colorimetric measurement of L-lactate concentration from apical secretions of *HK2*-overexpressing CF AECs, 72 h post-transfection. For all experiments n *≥* 3. Data are presented as mean *±* SEM. ***p < 0.001.
(TIFF)

## References

[1] McCullers Jonathan A. The co-pathogenesis of influenza viruses with bacteria in the lung. Nat Rev Microbiol. 2014 Apr 3;12(4):252–62. doi: 10.1038/nrmicro3231 24590244

[2] Oliva J, Terrier O. Viral and Bacterial Co-Infections in the Lungs: Dangerous Liaisons. Viruses. 2021 Aug 30;13(9). doi: 10.3390/v13091725 34578306 PMC8472850

[3] Petersen N T, Høiby N, Mordhorst C H, Lind K, Flensborg E W, Bruun B. Respiratory infections in cystic fibrosis patients caused by virus, chlamydia and mycoplasma–possible synergism with *Pseudomonas aeruginosa*. Acta Paediatr. 1981;70(5):623–8. 10.1111/j.1651-2227.1981.tb05757.x 6798822

[4] Smyth AR, Smyth RL, Tong CY, Hart CA, Heaf DP. Effect of respiratory virus infections including rhinovirus on clinical status in cystic fibrosis. Arch Dis Child. 1995 Aug 1;73(2). doi: 10.1136/adc.73.2.117 7574853 PMC1511210

[5] Collinson J, Nicholson KG, Cancio E, Ashman J, Ireland DC, Hammersley V, et al. Effects of upper respiratory tract infections in patients with cystic fibrosis. Thorax. 1996 Nov 1;51(11). doi: 10.1136/thx.51.11.1115 8958895 PMC1090523

[6] Thorburn K, Harigopal S, Reddy V, Taylor N, van Saene H K F. High incidence of pulmonary bacterial co-infection in children with severe respiratory syncytial virus (RSV) bronchiolitis. Thorax. 2006 Jul 1;61(7):611–5. doi: 10.1136/thx.2005.048397 16537670 PMC2104657

[7] Wark PAB, Tooze M, Cheese L, Whitehead B, Gibson PG, Wark KF, et al. Viral infections trigger exacerbations of cystic fibrosis in adults and children. Vol. 40, European Respiratory Journal. 2012. p. 510–2. doi: 10.1183/09031936.00202311 22855475

[8] DeMuri GP, Gern JE, Eickhoff JC, Lynch S V, Wald ER. Dynamics of Bacterial Colonization With *Streptococcus pneumoniae*, *Haemophilus influenzae*, *and Moraxella catarrhalis* During Symptomatic and Asymptomatic Viral Upper Respiratory Tract Infection. Clinical Infectious Diseases. 2018 Mar 19;66(7):1045–53. 29121208 10.1093/cid/cix941 PMC6019034

[9] Hedberg P, Johansson N, Ternhag A, Abdel-Halim L, Hedlund J, Nauclér P. Bacterial co-infections in community-acquired pneumonia caused by SARS-CoV-2, influenza virus and respiratory syncytial virus. BMC Infect Dis. 2022 Dec 31;22(1):1–11. 35100984 10.1186/s12879-022-07089-9 PMC8802536

[10] Peltola VT, Boyd KL, McAuley JL, Rehg JE, McCullers JA. Bacterial Sinusitis and Otitis Media following Influenza Virus Infection in Ferrets. Infect Immun. 2006 May;74(5):2562–7. doi: 10.1128/IAI.74.5.2562-2567.2006 16622191 PMC1459735

[11] Calvo C, Gallardo P, Torija P, Bellón S, Méndez-Echeverría A, del Rosal T, et al. Enterovirus neurological disease and bacterial coinfection in very young infants with fever. Journal of Clinical Virology. 2016 Dec;85:37–9. doi: 10.1016/j.jcv.2016.10.020 27833059

[12] Grimprel E, Rodrigo C, Desselberger U. Rotavirus Disease. Pediatric Infectious Disease Journal. 2008 Jan;27(1):3–10. 10.1097/01.inf.0000197563.03895.91 16397425

[13] Gonzalez-Galan V, Sánchez-Fauqier A, Obando I, Montero V, Fernandez M, Torres MJ, et al. High prevalence of community-acquired norovirus gastroenteritis among hospitalized children: a prospective study. Clinical Microbiology and Infection. 2011 Dec;17(12):1895–9. doi: 10.1111/j.1469-0691.2011.03506.x 21848976

[14] Valentini D, Vittucci AC, Grandin A, Tozzi AE, Russo C, Onori M, et al. Coinfection in acute gastroenteritis predicts a more severe clinical course in children. European Journal of Clinical Microbiology & Infectious Diseases. 2013 Jul 31;32(7):909–9015. doi: 10.1007/s10096-013-1825-9 23370970

[15] Mathew S, Smatti MK, Al Ansari K, Nasrallah GK, Al Thani AA, Yassine HM. Mixed Viral-Bacterial Infections and Their Effects on Gut Microbiota and Clinical Illnesses in Children. Sci Rep. 2019 Jan 29;9(1):1–12. 30696865 10.1038/s41598-018-37162-w PMC6351549

[16] Finelli L, Fiore A, Dhara R, Brammer L, Shay DK, Kamimoto L, et al. Influenza-Associated Pediatric Mortality in the United States: Increase of *Staphylococcus aureus* Coinfection. Pediatrics. 2008 Oct 1;122(4):805–11. 18829805 10.1542/peds.2008-1336

[17] Thors V, Christensen H, Morales-Aza B, Oliver E, Sikora P, Vipond I, et al. High-density Bacterial Nasal Carriage in Children Is Transient and Associated With Respiratory Viral Infections—Implications for Transmission Dynamics. Pediatric Infectious Disease Journal. 2019 May;38(5):533–8. doi: 10.1097/INF.0000000000002256 30985547

[18] Godefroy R, Giraud-gatineau A, Jimeno MT, Edouard S, Meddeb L, Zandotti C, et al. Respiratory Syncytial Virus Infection: Its Propensity for Bacterial Coinfection and Related Mortality in Elderly Adults. Open Forum Infect Dis. 2020 Dec 1;7(12):ofaa546. doi: 10.1093/ofid/ofaa546 33335940 PMC7733236

[19] Liu Y, Ling L, Wong SH, Wang MH, Fitzgerald JR, Zou X, et al. Outcomes of respiratory viral-bacterial co-infection in adult hospitalized patients. EClinicalMedicine. 2021 Jul;37:100955. doi: 10.1016/j.eclinm.2021.100955 34386745 PMC8343259

[20] Nakamura S, Davis KM, Weiser JN. Synergistic stimulation of type I interferons during influenza virus coinfection promotes *Streptococcus pneumoniae* colonization in mice. Journal of Clinical Investigation. 2011 Sep 1;121(9):3657–65. 21841308 10.1172/JCI57762 PMC3163966

[21] Jochems SP, Marcon F, Carniel BF, Holloway M, Mitsi E, Smith E, et al. Inflammation induced by influenza virus impairs human innate immune control of pneumococcus. Nat Immunol. 2018 Dec 29;19(12):1299–308. doi: 10.1038/s41590-018-0231-y 30374129 PMC6241853

[22] Hendricks MR, Lashua LP, Fischer DK, Flitter BA, Eichinger KM, Durbin JE, et al. Respiratory syncytial virus infection enhances *Pseudomonas aeruginosa* biofilm growth through dysregulation of nutritional immunity. Proc Natl Acad Sci U S A. 2016;113(6):1642–7. 26729873 10.1073/pnas.1516979113 PMC4760822

[23] Hendricks MR, Lane S, Melvin JA, Ouyang Y, Stolz DB, Williams J V., et al. Extracellular vesicles promote transkingdom nutrient transfer during viral-bacterial co-infection. Cell Rep. 2021 Jan;34(4). doi: 10.1016/j.celrep.2020.108672 33503419 PMC7918795

[24] Schoggins JW, Rice CM. Interferon-stimulated genes and their antiviral effector functions. Curr Opin Virol [Internet]. 2011 Dec;1(6):519–25. doi: 10.1016/j.coviro.2011.10.008 22328912 PMC3274382

[25] Schoggins JW. Interferon-Stimulated Genes: What Do They All Do? Annu Rev Virol. 2019 Sep 29;6(1):567–84. doi: 10.1146/annurev-virology-092818-015756 31283436

[26] Schoggins JW, Wilson SJ, Panis M, Murphy MY, Jones CT, Bieniasz P, et al. A diverse range of gene products are effectors of the type I interferon antiviral response. Nature. 2011 Apr 28;472(7344):481–5. doi: 10.1038/nature09907 21478870 PMC3409588

[27] Schoggins JW, MacDuff DA, Imanaka N, Gainey MD, Shrestha B, Eitson JL, et al. Pan-viral specificity of IFN-induced genes reveals new roles for cGAS in innate immunity. Nature. 2014 Jan 30;505(7485):691–5. doi: 10.1038/nature12862 24284630 PMC4077721

[28] Lunt SY, Vander Heiden MG. Aerobic glycolysis: Meeting the metabolic requirements of cell proliferation. Annu Rev Cell Dev Biol. 2011;27:441–64. doi: 10.1146/annurev-cellbio-092910-154237 21985671

[29] Schulze A, Harris AL. How cancer metabolism is tuned for proliferation and vulnerable to disruption. Vol. 491, Nature. 2012. p. 364–73. doi: 10.1038/nature11706 23151579

[30] Patra KC, Wang Q, Bhaskar PT, Miller L, Wang Z, Wheaton W, et al. Hexokinase 2 Is Required for Tumor Initiation and Maintenance and Its Systemic Deletion Is Therapeutic in Mouse Models of Cancer. Cancer Cell. 2013 Aug;24(2):213–28. doi: 10.1016/j.ccr.2013.06.014 23911236 PMC3753022

[31] Blaha CS, Ramakrishnan G, Jeon SM, Nogueira V, Rho H, Kang S, et al. A non-catalytic scaffolding activity of hexokinase 2 contributes to EMT and metastasis. Nat Commun. 2022 Feb 16;13(1):899. doi: 10.1038/s41467-022-28440-3 35173161 PMC8850586

[32] Vander Heiden MG, Cantley LC, Thompson CB. Understanding the Warburg Effect: The Metabolic Requirements of Cell Proliferation. Science (1979). 2009 May 22;324(5930):1029–33. doi: 10.1126/science.1160809 19460998 PMC2849637

[33] Empey KM, Orend JG, Peebles RS, Egaña L, Norris KA, Oury TD, et al. Stimulation of immature lung macrophages with intranasal interferon gamma in a novel neonatal mouse model of respiratory syncytial virus infection. PLoS One. 2012;7(7):e40499. doi: 10.1371/journal.pone.0040499 22792355 PMC3391240

[34] Moreau-Marquis S, Bomberger JM, Anderson GG, Swiatecka-Urban A, Ye S, O’Toole GA, et al. The ΔF508-CFTR mutation results in increased biofilm formation by *Pseudomonas aeruginosa* by increasing iron availability. American Journal of Physiology-Lung Cellular and Molecular Physiology. 2008 Jul;295(1):L25–37. 18359885 10.1152/ajplung.00391.2007 PMC2494796

[35] Zemke AC, Shiva S, Burns JL, Moskowitz SM, Pilewski JM, Gladwin MT, et al. Nitrite modulates bacterial antibiotic susceptibility and biofilm formation in association with airway epithelial cells. Free Radic Biol Med. 2014;77:307–16. doi: 10.1016/j.freeradbiomed.2014.08.011 25229185 PMC4278422

[36] Perelman SS, Abrams ME, Eitson JL, Chen D, Jimenez A, Mettlen M, et al. Cell-Based Screen Identifies Human Interferon-Stimulated Regulators of *Listeria monocytogenes* Infection. PLoS Pathog. 2016;12(12):e1006102. 28002492 10.1371/journal.ppat.1006102 PMC5176324

[37] Ren L, Zhang W, Zhang J, Zhang J, Zhang H, Zhu Y, et al. Influenza A Virus (H1N1) Infection Induces Glycolysis to Facilitate Viral Replication. Virol Sin. 2021 Dec 14;36(6):1532–42. doi: 10.1007/s12250-021-00433-4 34519916 PMC8692537

[38] Morris DR, Qu Y, Agrawal A, Garofalo RP, Casola A. HIF-1α Modulates Core Metabolism and Virus Replication in Primary Airway Epithelial Cells Infected with Respiratory Syncytial Virus. Viruses. 2020 Sep 26;12(10):1088. 32993138 10.3390/v12101088 PMC7601280

[39] Zhao Y, Chahar HS, Komaravelli N, Dossumbekova A, Casola A. Human metapneumovirus infection of airway epithelial cells is associated with changes in core metabolic pathways. Virology. 2019 May;531:183–91. doi: 10.1016/j.virol.2019.03.011 30927711 PMC6486412

[40] Thai M, Graham NA, Braas D, Nehil M, Komisopoulou E, Kurdistani SK, et al. Adenovirus E4ORF1-Induced MYC Activation Promotes Host Cell Anabolic Glucose Metabolism and Virus Replication. Cell Metab. 2014 Apr;19(4):694–701. doi: 10.1016/j.cmet.2014.03.009 24703700 PMC4294542

[41] Michi AN, Yipp BG, Dufour A, Lopes F, Proud D. PGC-1α mediates a metabolic host defense response in human airway epithelium during rhinovirus infections. Nat Commun. 2021 Dec 16;12(1):3669. 34135327 10.1038/s41467-021-23925-z PMC8209127

[42] Zhang W, Wang G, Xu ZG, Tu H, Hu F, Dai J, et al. Lactate Is a Natural Suppressor of RLR Signaling by Targeting MAVS. Cell. 2019 Jun;178(1):176–189.e15. doi: 10.1016/j.cell.2019.05.003 31155231 PMC6625351

[43] Pohanka M. D-Lactic Acid as a Metabolite: Toxicology, Diagnosis, and Detection. Biomed Res Int. 2020 Jun 18;2020:3419034. doi: 10.1155/2020/3419034 32685468 PMC7320276

[44] Fredman G, Kolpen M, Hertz FB, Petersen PT, Jensen AV, Baunbaek-Egelund G, et al. The inflamed sputum in lower respiratory tract infection: L-lactate levels are correlated to neutrophil accumulation. APMIS. 2019 Feb;127(2):72–9. doi: 10.1111/apm.12913 30614067 PMC7159756

[45] Palsson-McDermott EM O’Neill LAJ. The Warburg effect then and now: From cancer to inflammatory diseases. BioEssays. 2013 Nov;35(11):965–73. 24115022 10.1002/bies.201300084

[46] Bensel T, Stotz M, Borneff-Lipp M, Wollschläger B, Wienke A, Taccetti G, et al. Lactate in cystic fibrosis sputum. Journal of Cystic Fibrosis. 2011 Jan;10(1). doi: 10.1016/j.jcf.2010.09.004 20947455

[47] Exley RM, Goodwin L, Mowe E, Shaw J, Smith H, Read RC, et al. *Neisseria meningitidis* lactate permease is required for nasopharyngeal colonization. Infect Immun. 2005;73(9):5762–6. 16113293 10.1128/IAI.73.9.5762-5766.2005 PMC1231078

[48] Sigurlásdóttir S, Engman J, Eriksson OS, Saroj SD, Zguna N, Lloris-Garcerá P, et al. Host cell-derived lactate functions as an effector molecule in Neisseria meningitidis microcolony dispersal. PLoS Pathog. 2017;13(4):e1006251. doi: 10.1371/journal.ppat.1006251 28384279 PMC5383330

[49] Hosmer J, Nasreen M, Dhouib R, Essilfie AT, Schirra HJ, Henningham A, et al. Access to highly specialized growth substrates and production of epithelial immunomodulatory metabolites determine survival of *Haemophilus influenzae* in human airway epithelial cells. PLoS Pathog. 2022;18(1):e1010209. 35085362 10.1371/journal.ppat.1010209 PMC8794153

[50] Palmer KL, Aye LM, Whiteley M. Nutritional cues control *Pseudomonas aeruginosa* multicellular behavior in cystic fibrosis sputum. J Bacteriol. 2007;189(22):8079–87. 17873029 10.1128/JB.01138-07 PMC2168676

[51] La Rosa R, Johansen HK, Molina S. Convergent metabolic specialization through distinct evolutionary paths in Pseudomonas aeruginosa. mBio. 2018;9(2):e00269–18. doi: 10.1128/mBio.00269-18 29636437 PMC5893872

[52] Phan J, Gallagher T, Oliver A, England WE, Whiteson K. Fermentation products in the cystic fibrosis airways induce aggregation and dormancy-associated expression profiles in a CF clinical isolate of *Pseudomonas aeruginosa*. FEMS Microbiol Lett. 2018;365(10):fny082. 29617986 10.1093/femsle/fny082 PMC5928460

[53] Lin YC, Cornell WC, Jo J, Price-Whelan A, Dietrich LEP. The *Pseudomonas aeruginosa* Complement of Lactate Dehydrogenases Enables Use of D- and L-Lactate and Metabolic Cross-Feeding. mBio. 2018 Nov 7;9(5):e00961–18. 30206167 10.1128/mBio.00961-18 PMC6134097

[54] Wang Y, Xiao D, Liu Q, Zhang Y, Hu C, Sun J, et al. Two NAD-independent l-lactate dehydrogenases drive l-lactate utilization in *Pseudomonas aeruginosa* PAO1. Environ Microbiol Rep. 2018;10(5):569–75. 30066495 10.1111/1758-2229.12666

[55] Yung YP, McGill SL, Chen H, Park H, Carlson RP, Hanley L. Reverse diauxie phenotype in *Pseudomonas aeruginosa* biofilm revealed by exometabolomics and label-free proteomics. NPJ Biofilms Microbiomes. 2019;5(1):31. 31666981 10.1038/s41522-019-0104-7 PMC6814747

[56] Sajjan US, Jia Y, Newcomb DC, Bentley JK, Lukacs NW, LiPuma JJ, et al. H. influenzae potentiates airway epithelial cell responses to rhinovirus by increasing ICAM-1 and TLR3 expression. The FASEB Journal. 2006 Oct 16;20(12):2121–3. doi: 10.1096/fj.06-5806fje 16914605

[57] Verkaik NJ, Nguyen DT, de Vogel CP, Moll HA, Verbrugh HA, Jaddoe VWV, et al. *Streptococcus pneumoniae* exposure is associated with human metapneumovirus seroconversion and increased susceptibility to in vitro HMPV infection. Clinical Microbiology and Infection. 2011 Dec;17(12):1840–4. 21883660 10.1111/j.1469-0691.2011.03480.x

[58] Kuss SK, Best GT, Etheredge CA, Pruijssers AJ, Frierson JM, Hooper L V., et al. Intestinal Microbiota Promote Enteric Virus Replication and Systemic Pathogenesis. Science (1979). 2011 Oct 14;334(6053):249–52. doi: 10.1126/science.1211057 21998395 PMC3222156

[59] Rowe HM, Livingston B, Margolis E, Davis A, Meliopoulos VA, Echlin H, et al. Respiratory Bacteria Stabilize and Promote Airborne Transmission of Influenza A Virus. mSystems. 2020 Oct 27;5(5). doi: 10.1128/mSystems.00762-20 32873612 PMC7470989

[60] Bomberger JM, Ely KH, Bangia N, Ye S, Green KA, Green WR, et al. *Pseudomonas aeruginosa* Cif Protein Enhances the Ubiquitination and Proteasomal Degradation of the Transporter Associated with Antigen Processing (TAP) and Reduces Major Histocompatibility Complex (MHC) Class I Antigen Presentation. Journal of Biological Chemistry. 2014 Jan;289(1):152–62. 24247241 10.1074/jbc.M113.459271 PMC3879540

[61] Wang S, El-Deiry WS. TRAIL and apoptosis induction by TNF-family death receptors. Oncogene. 2003 Nov 24;22(53):8628–33. doi: 10.1038/sj.onc.1207232 14634624

[62] Regad T, Chelbi-Alix MK. Role and fate of PML nuclear bodies in response to interferon and viral infections. Oncogene. 2001 Oct 29;20(49):7274–86. doi: 10.1038/sj.onc.1204854 11704856

[63] Deguine J, Barton GM. MyD88: a central player in innate immune signaling. F1000Prime Rep. 2014 Nov 4;6. doi: 10.12703/P6-97 25580251 PMC4229726

[64] Naslavsky N, Caplan S. EHD proteins: key conductors of endocytic transport. Trends Cell Biol. 2011 Feb;21(2):122–31. doi: 10.1016/j.tcb.2010.10.003 21067929 PMC3052690

[65] Roberts DJ, Miyamoto S. Hexokinase II integrates energy metabolism and cellular protection: Akting on mitochondria and TORCing to autophagy. Cell Death Differ. 2015 Feb 17;22(2):248–57. doi: 10.1038/cdd.2014.173 25323588 PMC4291497

[66] Bomberger Jennifer. Interferon stimulated genes induce Pseudomonas aeruginosa biofilms through metabolic reprogramming [Dataset]. Dryad. doi: 10.5061/dryad.djh9w0w5v

