# Supplementary figures and images for: Interferon signaling drives epithelial metabolic reprogramming to promote secondary bacterial infection
Source: PLoS Pathog. 2023 Nov 8;19(11):e1011719. doi: 10.1371/journal.ppat.1011719 (PMC10631704; doi:10.1371/journal.ppat.1011719)

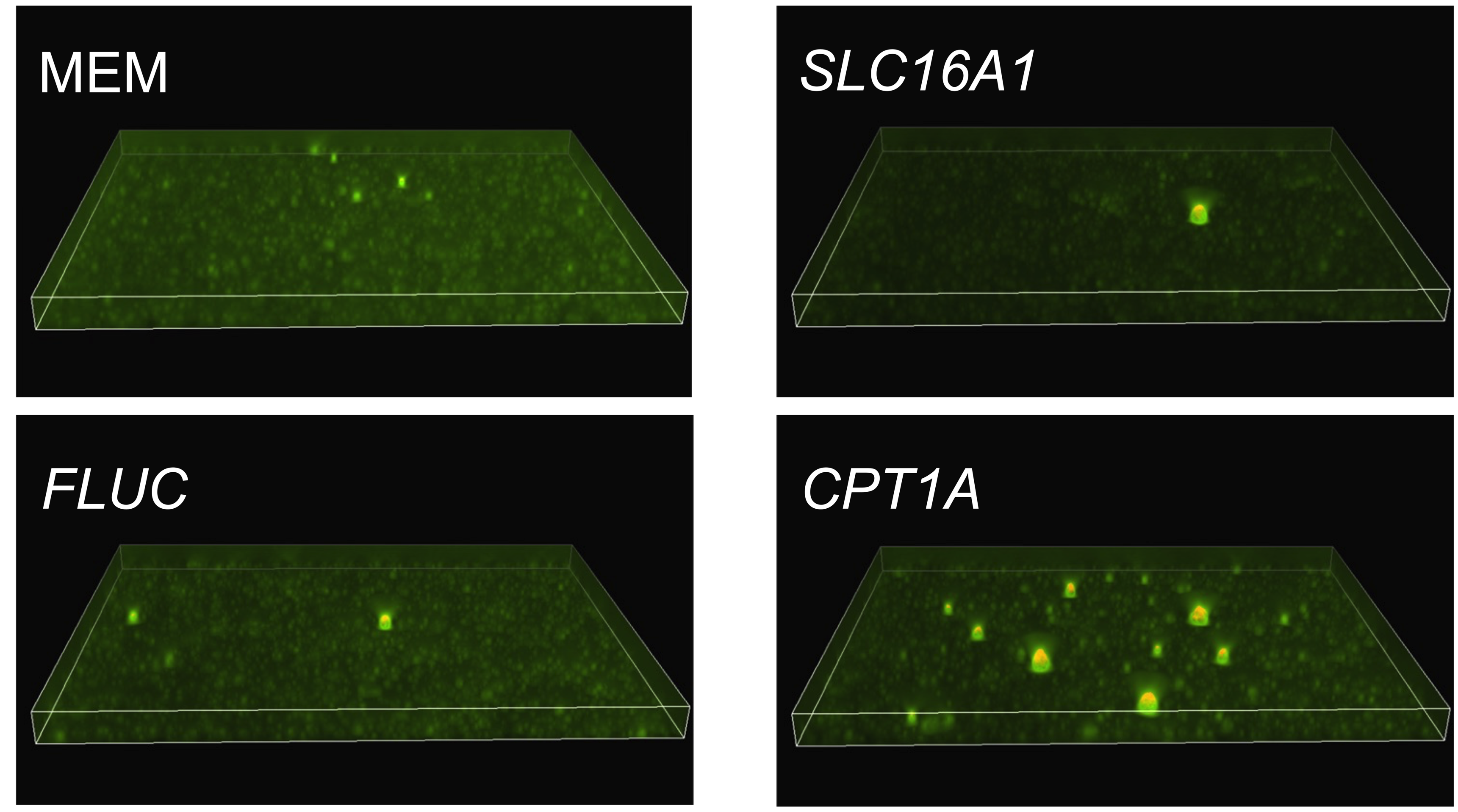

Supplement: S1 Fig — Representative images of GFP-producing P. aeruginosa biofilms under abiotic conditions grown in MEM (negative control), secretions from cells overexpressing FLUC (transduction control), and secretions from cells transfected with an ISG that resulted in low biofilm formation induction (SLC16A1) and an ISG that resulted in high biofilm formation induction (CPT1A). 20x images captured with Nikon NIS-Elements. (TIF) [file ppat.1011719.s001.tif]

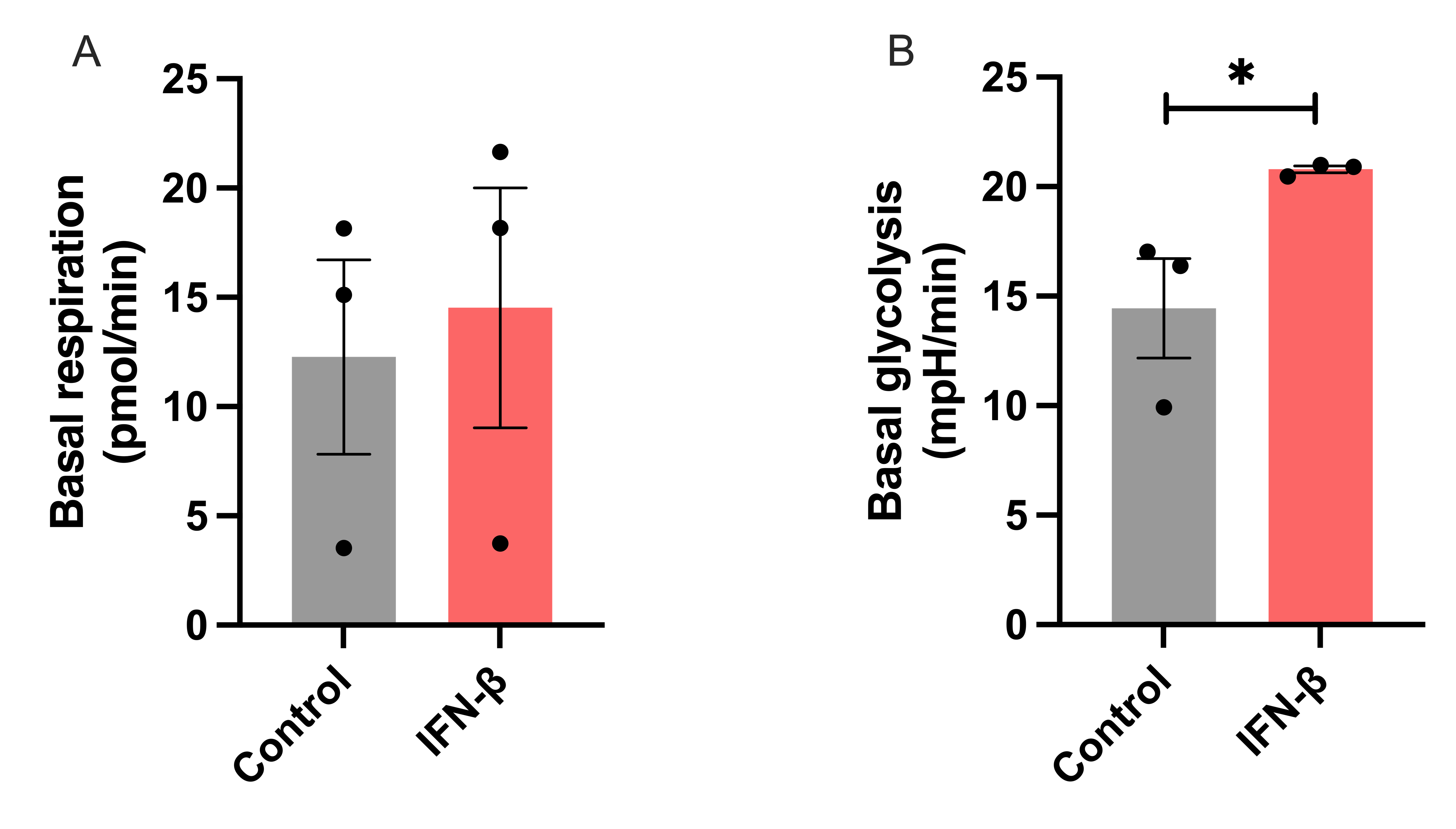

Supplement: S2 Fig — Measurement of (A) basal respiration and (B) basal glycolysis in CFBE41o- stimulated with IFN-β (1000 IU/mL) using a Seahorse assay. For all experiments n ≥ 3. Data are presented as mean ± SEM. *p < 0.05. (TIF) [file ppat.1011719.s002.tif]

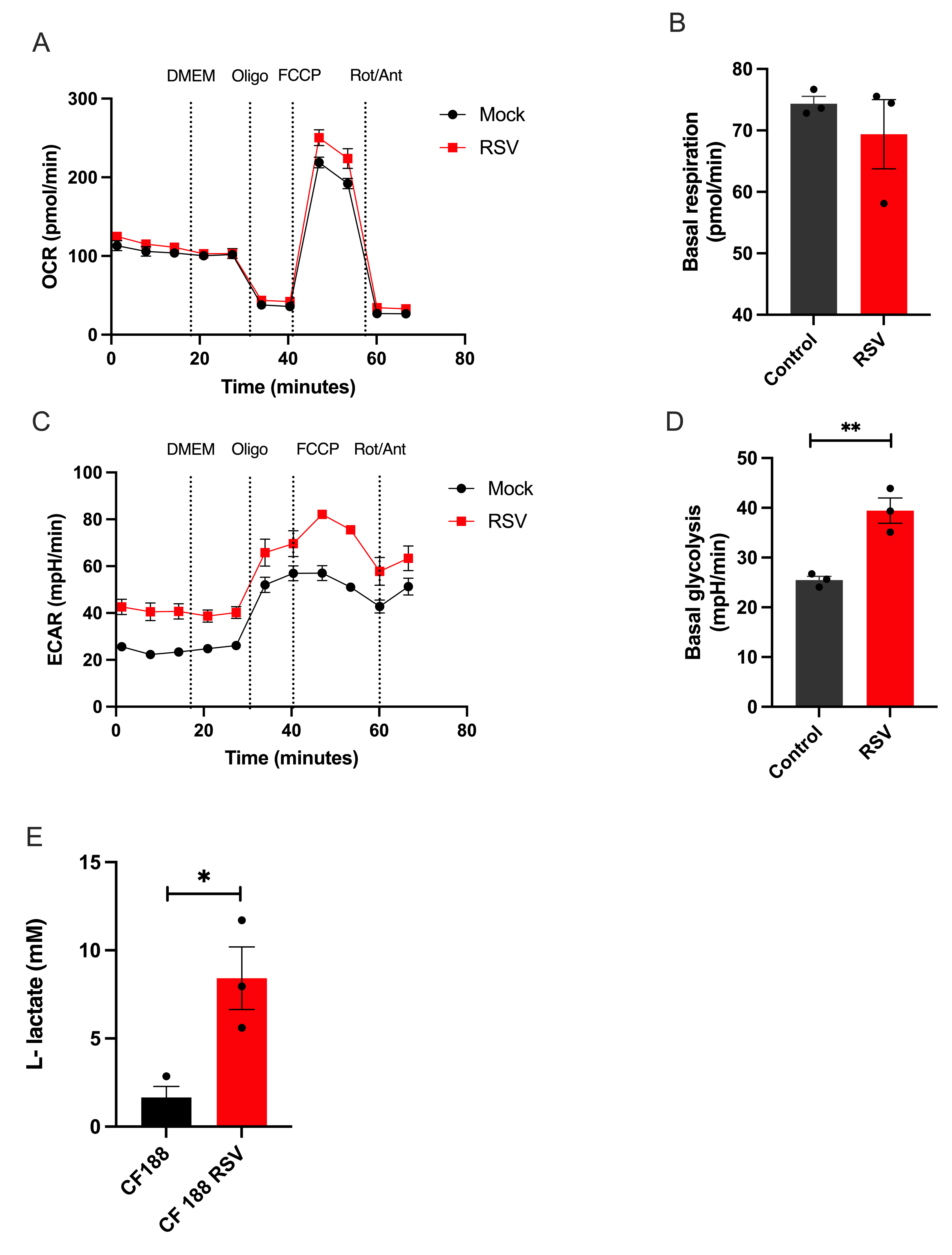

Supplement: S3 Fig — (A) OCR, (B) basal respiration, (C) ECAR, and (D) basal glycolysis measured in RSV-infected CFBE41o- cells using a Seahorse assay. (E) L-lactate concentration in apical secretions from primary CF AECs during RSV infection measured by colorimetric assay. For all experiments n ≥ 3. Data are presented as mean ± SEM. *p < 0.05, **p < 0.01. (TIF) [file ppat.1011719.s003.tif]

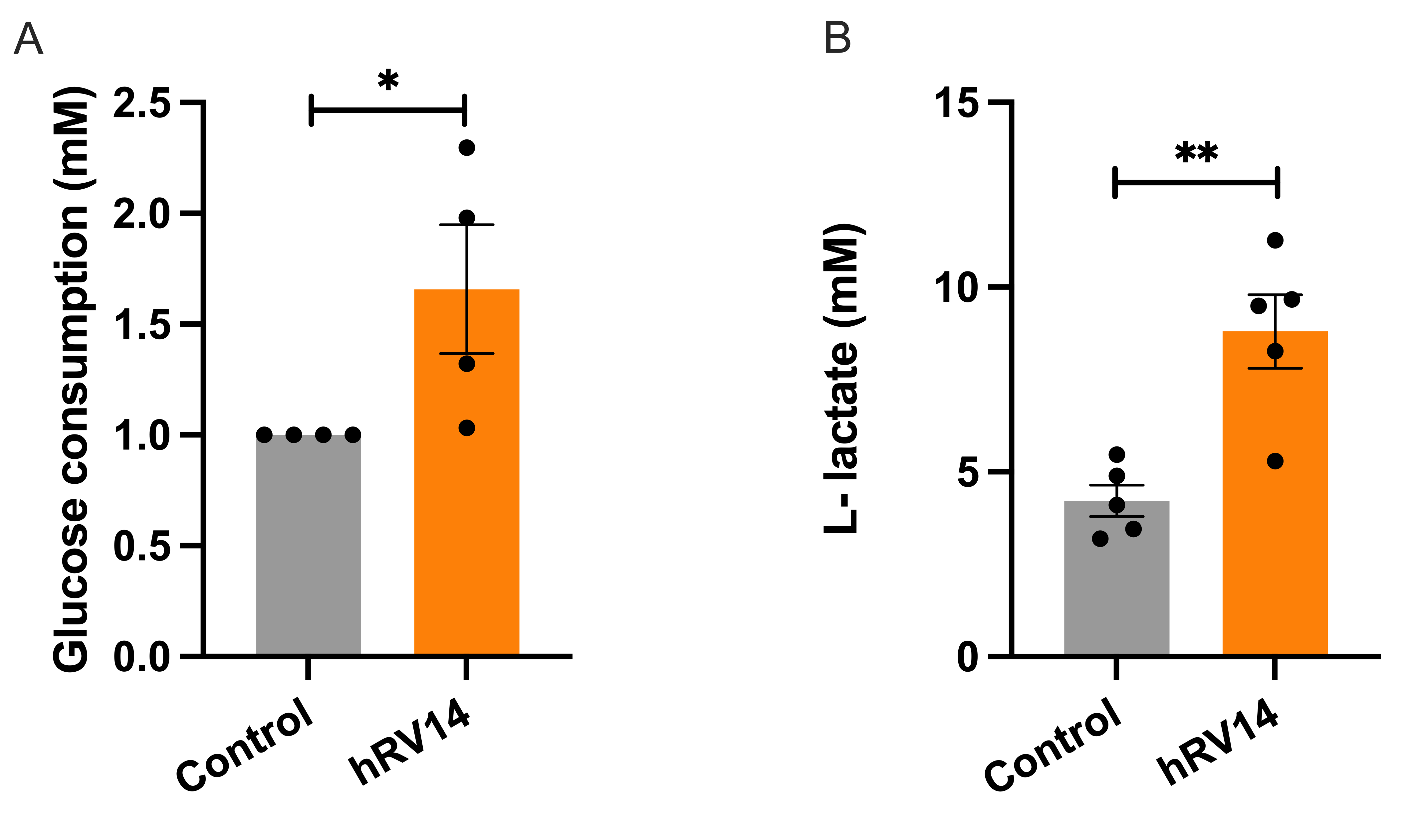

Supplement: S4 Fig — Measurement of (A) glucose consumption in growth medium and (B) L-lactate concentration in apical secretions of CF AECs infected with hRV14 (MOI 0.1) for 72 h using a colorimetric assay. For all experiments n ≥ 3. Data are presented as mean ± SEM. *p < 0.05, **p < 0.01. (TIF) [file ppat.1011719.s004.tif]

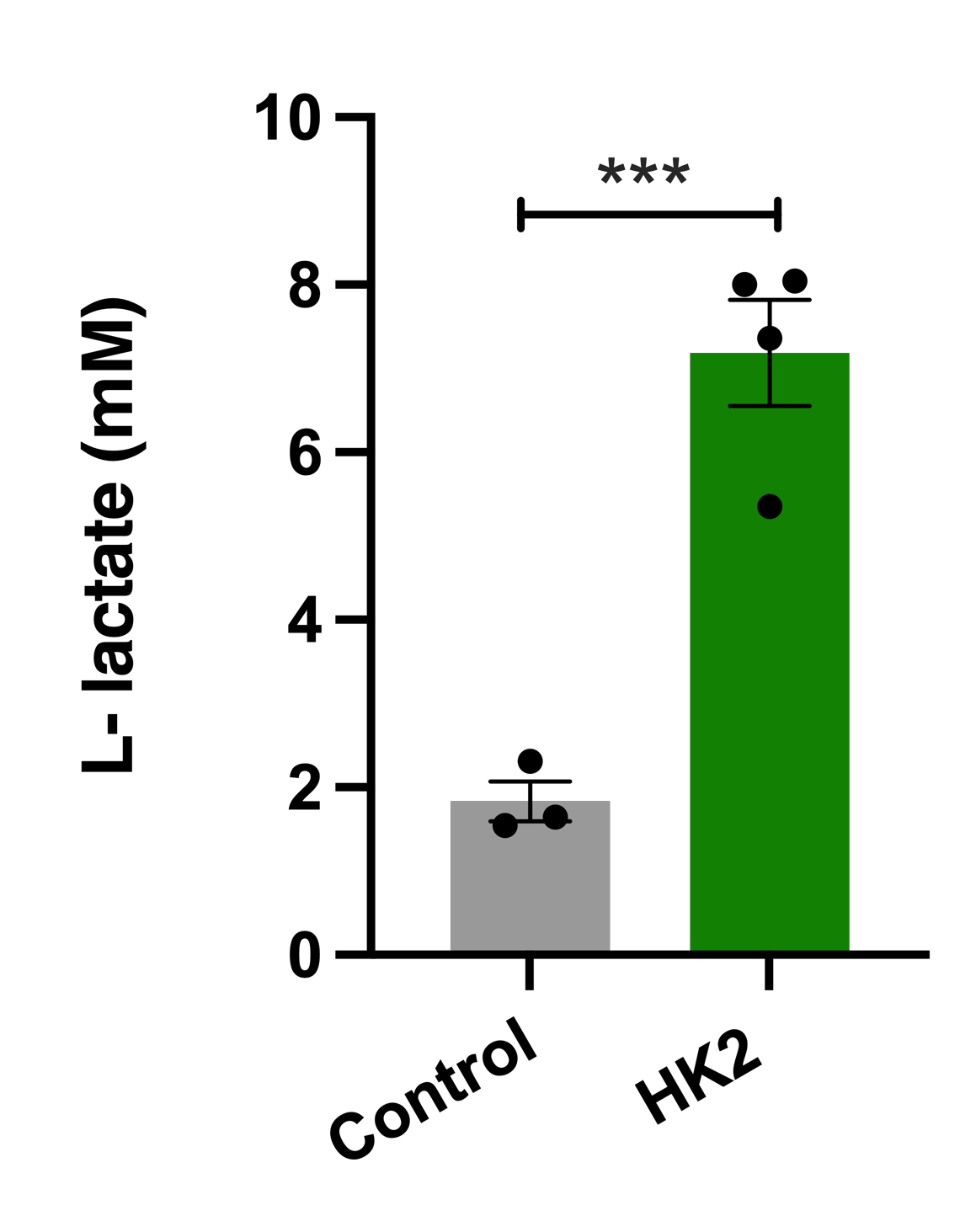

Supplement: S5 Fig — Colorimetric measurement of L-lactate concentration from apical secretions of HK2-overexpressing CF AECs, 72 h post-transfection. For all experiments n ≥ 3. Data are presented as mean ± SEM. ***p < 0.001. (TIFF) [file ppat.1011719.s005.tiff]
